# Gray Matter Abnormalities in Schizophrenia Patients with Tardive Dyskinesia: A Magnetic Resonance Imaging Voxel-Based Morphometry Study

**DOI:** 10.1371/journal.pone.0071034

**Published:** 2013-08-15

**Authors:** Cheng-Ta Li, Kun-Hsien Chou, Tung-Ping Su, Chu-Chung Huang, Mu-Hong Chen, Ya-Mei Bai, Ching-Po Lin

**Affiliations:** 1 Institute of Neuroscience, National Yang-Ming University, Taipei, Taiwan; 2 Department of Psychiatry, Taipei Veterans General Hospital, Taipei, Taiwan; 3 Institute of Brain Science, National Yang-Ming University, Taipei, Taiwan; 4 Division of Psychiatry, Faculty of Medicine, National Yang-Ming University, Taipei, Taiwan; 5 Department of Biomedical Imaging and Radiological Sciences, National Yang-Ming University, Taipei, Taiwan; Beijing Normal University, China

## Abstract

**Objective:**

The pathophysiological mechanism of TD remains unknown. All previous studies, using the region-of-interest method, focused on basal ganglion areas, were with inconsistent results. This whole-brain voxel-based morphometry (VBM) study investigate the grey matter abnormality of TD and its correlates with clinical ratings.

**Method:**

High resolution T1-weighted brain volumetric MRI from 25 schizophrenia patients with TD (TD group), 25 age-, gender-, and handedness-matched schizophrenia patients without TD (non-TD group), and 25 matched healthy subjects (NC group) were analyzed using a VBM approach. Clinical ratings included the Positive and Negative Symptom Scale (PANSS), Abnormal Involuntary Movement Scale (AIMS), and the Simpson-Angus Scale (SAS).

**Results:**

The TD group had significantly smaller total gray matter volumes than the NC group (p = 0.05). Compared to the non-TD group, the TD group had significantly higher PANSS negative (p<0.001), SAS (p<0.001), and AIMS (p<0.001) scores; and smaller bilateral inferior frontal gyrus, which correlated negatively with the PANSS negative scores (r = −0.366, p<0.05); and smaller right superior frontal gyrus, which correlated negatively with AIMS scores (r = −0.399, p<0.001), and PANSS general score (r = −0.338, p<0.05).

**Limitations:**

The cross-section design can’t separate the gray matter change to TD from the context of the illness of schizophrenia, although TD with more severe clinical psychopathology could be a phenotype.

**Conclusions:**

The schizophrenia patients with TD had significantly reduced gray matter, mostly at the bilateral inferior frontal gyrus and the right superior frontal gyrus, which correlated with severity of clinical symptoms and involuntary movement, respectively.

## Introduction

Tardive dyskinesia (TD), a severe and disabling side effect of antipsychotics, is characterized by late-onset, repetitive, involuntary choreiform movements, tics and grimaces of the orofacial muscles, and dyskinesia of the distal limbs, paraspinal muscles, and diaphragm [1]; which may cause appearance deformity, daily function disability, and even legal sues [2], [3]. More than half of TD cases may persist, even after conventional antipyschotics are switched to atypical antipsychotics [4] or antipsychotics are discontinued [5]. There has been less attention focused on TD for the past few years since the development of atypical antipsychotics, which is regarded to have lowered the risk of TD. But actually the annualized incidence of TD with atypical antipsychotics was still up to 3.9% [6], [7]; although this was lower than that of conventional antipsychotics (5.5%), it was higher than expected. Woods SW et al found adjusted tardive dyskinesia incidence rate-ratio for subjects treated with atypical antipsychotics alone was 0.68 (95% CI, 0.29–1.64) compared to conventional antipsychotics by following 352 patients for 4 years [8]. Therefore, gaining an understanding of the pathophysiology of TD is still important [9].

Basal ganglia and nigro-striatal pathway play important roles in movement control, and has long been a target of interest while investigating underlying brain pathophysiology for TD. However, previous studies, all using the region-of-interest (ROI) method, focused on basal ganglion areas, were with inconsistent results. Bartzokis and Granholm et al found that schizophrenia patients with TD had significantly shortened left caudate T2 relaxation times, compared to patients without TD [10], [11]. Mion et al found the volumes of the caudate nuclei of patients with TD were significantly smaller than those of patients without TD and normal controls [12]. However, Elkashef et al. failed to find significant differences in the volume of globus pallidus and putamen between patients with and without persistent TD [13]. Buckley P et al found schizophrenia patients showed more prolonged T2 relaxation times in the right putamen and globus pallidus than did control subjects, but no significant difference in T2 values was found between patients with and without TD [14]. Harvey et al also didn’t find significant differences in the T1 relaxation time of the basal ganglia between schizophrenia patients with and without TD [15]. Thus, the previous results of neuroimaging studies investigating basal ganglia for TD were still controversial, and the pathophysiologic mechanism of TD may involve other brain areas.

Previous clinical studies had shown that schizophrenia patients with TD presented with more negative symptoms and cognitive dysfunction, both of which have close relationship with prefrontal cortex [16]–[18]. Negative symptoms in schizophrenia are widely accepted to reflect hypofrontality [19], [20]. However, these prefrontal cortex areas were not investigated in previous imaging studies of TD. Since all the previous studies were undertaken more than 15 years ago, the researchers only focused on striatal basal ganglion areas, may be due to the limitation of technique with ROI analysis methods at that time. Nowadays, using the Voxel-Based Morphometry (VBM) analysis method, the difference in the gray matter can be investigated in whole brain manner, instead of being focused only on specific ROIs. Recently, in a VBM study, Cerasa et al. reported gray matter alterations in the bilateral inferior frontal gyrus, but not in the basal ganglia, in patients with Parkinson’s disease with levodopa-induced dyskinesia compared to those without [21]. Therefore, whether drug-induced dyskinesia is resulted from cortical or subcortical tissue alterations needs further studies to clarify, because the most specific pathophysiological processes underlying movement disorders are still not completely understood [22].

In this study, using fully automated unbiased whole brain VBM approach, we compared the gray matter volume of schizophrenia patients with/without TD, and matched healthy subjects. We hypothesized that a greater reduction of gray matter volume in basal ganglia and prefrontal cortex would be found in schizophrenia patients with TD in areas involved in movement control and in clinical symptoms.

## Materials and Methods

### Sample collection and Clinical Assessments

The study subjects were 25 schizophrenia (*Diagnostic and Statistical Manual of Mental Disorders*, fourth edition, or *DSM-IV*) patients with TD and 25 without, and 25 matched healthy subjects (normal control group). These three groups were matched for age, gender ratio and handedness. Schooler and Kane’s Research Diagnostic Criteria were used to define TD: (1) moderate abnormal involuntary movement in one or more body area, or (2) mild involuntary movements in two or more areas [23]. The healthy control subjects were interviewed using the Mini-International Neuropsychiatric Interview (MINI) to confirm there was no previous history of neurologic or psychiatric illness, and all had a normal brain structure, as confirmed by MRI scans. Subjects were excluded if they had another Axis I psychiatric diagnosis, serious neurologic or endocrine disorders, any medical condition or treatment known to affect the brain, alcohol/substance misuse-related disorders, or mental retardation defined according to *DSM-IV* criteria. The clinical rating scales included the Positive and Negative Symptom Scale (PANSS) for severity of psychopathology, the Abnormal Involuntary Movement Scale (AIMS) for TD, and the Simpson-Angus Scale (SAS) for extrapyramidal side effects. The clinical ratings were performed by Dr. Bai, who has years of experience using AIMS ratings for TD [4], [5], [24]–[42]. All participants were accompanied with family relatives or care takers to assure they understood and provided the written informed consent before participating in the study. This study was approved by the local ethics committee of human research at Taipei Veterans General Hospital in Taiwan.

### MRI data acquisition

All brain images were acquired on a 1.5-T MR system (Excite II; GE Medical Systems, Milwaukee, Wis, USA) with eight-channel head coil. Three-dimensional fluid-attenuated inversion-recovery fast spoiled gradient recalled echo (3D FLAIR-FSPGR) sequence was applied to obtain the high resolution structural images in the axial plane with following imaging parameters: TR/TE/TI  = 8.548/1.836/400 ms; flip angle  = 15°; field of view  = 260×260 mm^2^; 124 slices; matrix size  = 256×256; NEX = 1; slice thickness  = 1.5 mm and voxel size  = 1.02×1.02×1.5 mm^3^. All the images were acquired parallel to the anterior commissure-posterior commissure line. To minimize motion artifact generated during the image acquisition, each subject’s head was immobilized with cushions inside the coil.

### DARTEL-based T1 Voxel-Based Morphometry

Individual high resolution T1-weighted volumetric images were analyzed using the Gaser’s VBM8 toolbox (http://dbm.neuro.uni-jena.de) with Statistical Parametric Mapping (SPM8, wellcome Institute of Neurology, University College London, UK) executed in Linux-based MATLAB 2010a (The MathWorks, Natick, MA, USA) platform with default settings. VBM8 toolbox which extended the original unified segmentation model included three preprocessing steps: (1) noise reduction (2) field inhomogeneity correction (3) tissue segmentation to further improve the accuracy of tissue segmentation. In this study, the detail VBM approach included the followings: Data were first carefully checked to be without any scanner artifacts, motion problems or gross anatomical abnormalities for each participant by experienced radiologist. After data checking and origin identification, noise reduction procedure was performed on each participant’s native space T1w structural image using the spatial adaptive non local means denoising filter. Then high signal-to-noise ratio T1w images were segmented into three tissue components (GM,WM and CSF) using an adaptive maxium a posteriori segmentation approach [43] with partial volume estimation technique [44] and further refined by applying an iterative hidden Markov field (HMRF) model [45] to remove isolated voxels which are unlikely to belong to a determinate tissue type and improve the quality of tissue segmentation. To achieve higher accuracy of registration across subjects, each native space tissue segments were imported into a rigidly aligned space and iteratively registered to group-specific templates which were generated from all structural images in this study through non-linear warping by using DARTEL toolbox. In order to preserve actual volumetric information, deformation parameters obtaining in previous step were applied to modulate the GM, WM and CSF tissue segments of participants. Since DARTEL worked with images with averaged brain size of total participants in this study, additional affine transformation between average group space and MNI (Montreal Neurological Institute) standard space was needed to achieve a suitable alignment between these two spaces. Finally, each modulated tissue segments were written with an isotropic voxel resolution of 1×1×1 mm^3^. All normalized, segmented, and modulated MNI standard space images were smoothed with an 8-mm Gaussian kernel before tissue volume calculation and voxel-wise group comparisons. Total intracranial volume (TIV) was determined as the sum of GM, WM and CSF volumes.

### Statistical analysis

The analysis of variance (ANOVA) with post-hoc Tukey correction and Chi-square test were applied to compare the continuous demographic variables and categorical data among three groups respectively. The regional gray matter volume of detected suprathreshold clusters were extracted for each participant from the contrast of the direct group comparison of two disease groups (schizophrenia without TD vs. schizophrenia with TD). Smoothed modulated gray matter segments were analyzed with SPM8 utilizing the framework of General Linear Model (GLM). Voxel-wise GM volume differences among three groups were investigated using Analysis of covariance (ANCOVA) model with co-varying the age, sex, former education years and TIV. To avoid possible edge effects around the margin between different tissue types, all voxels with a GM probability value lower than 0.2 (absolute threshold, range from 0 to 1) were excluded. As we had a prior hypothesis about the localization of structural difference (frontal/prefrontal and other motor control related brain regions), significant levels for each t statistics of whole brain-wise group comparison were set at uncorrected voxel-level p-value less than 0.001 with a spatial extent of 50 voxels. The command-line tool (i.e., 3dClusterSim) was used to correct for image-based multiple comparisons [46]–[49]. The 3dClusterSim is available in the AFNI toolbox (Analysis of Functional NeuroImages, http://afni.nimh.nih.gov/afni/). The statistical threshold for each voxel was set at corrected P_FWE-corrected_ <0.05, with a cluster size of at least 541 voxels, based on the results of the Monte Carlo simulation (3dClusterSim with the following parameters: single voxel p value  = 0.001, 10,000 simulations, FWHM  = 7.67 * 8.80 * 8.47 mm with GM mask). Here we considered both the results (P_uncorrected_ <0.001 and P_FWE-corrected_ <0.05) as statistically significant, in order to provide comprehensive information and to prevent from being over-conservative in the *a-priori* regions.

In addition to whole brain analysis, the basal ganglia was defined as the primary volume of interest (VOI) due to investigate inconsistent findings from previous studies [14].The VOIs were defined bilaterally using WFU-Pick atlas toolbox. Small volume correction (SVC) was applied within each basal ganglia VOIs to correct for multiple comparison problems. Voxel were assessed for significance using the family-wise error (FWE) corrected statistics for an appropriate correction (FWE-corrected p value <0.05). Voxels showing a statistical difference of uncorrected p-value <0.001 in the basal ganglia were also reported as trend changes. GingerALE toolbox (The BrainMap Development Team; http://brainmap.org/ale/index.html) was used to transform MNI coordinates into Talairach coordinates to account for minimizing coordinate transformation discrepancy between MNI and Talairach space. Anatomical structures of the coordinates representing significant clusters were identified on the basis of the Talairach and Tournoux atlas [50]. To clarify the neuroanatomical correlates of individual differences in clinical evaluations, partial Pearson correlation analysis with age, sex, former education years and total intracranial volume (TIV) as confounding covariates was performed to correlate the PANSS, AIMS and SAS scores with the regional GM volume within patient group. The threshold was first set at P<0.05 for an exploratory purpose, but considering the issue of multiple comparisons, the threshold for statistical significance was set at P<0.0125 (0.05/4, corrected for the 4 detected suprathreshold clusters). Finally, to determine the most important factors accounting for the GM volume changes in schizophrenia with TD, stepwise multiple regression analysis was used to reveal the association between clinical and demographic variables with regional GM volumes. Age, sex, education level, TIV, PANSS scores (Total, Negative, Positive, and General), AIMS, and SAS scores were entered as predictors. All data processing and statistical analyses were performed with Statistical Package for Social Science (SPSS) version 17 software (SPSS Inc).

## Results

### Characteristics and global tissue volume results of the participants

In total, 25 schizophrenia patients with TD, 25 patients without TD and 25 age- and gender-matched normal controls were enrolled. There was no significant difference in the age at onset of illness, duration of illness, or antipsychotic chlorpromazine equivalent dose between the two groups of schizophrenia subjects; who all received only one antipsychotic drug. Compared with the Schizophrenia without TD group, the Schizophrenia with TD group had significantly higher PANSS total (p = 0.003), negative (p<0.001), general psychopathology scale scores (p = 0.012), AIMS (p<0.001) and SAS scores (p<0.001). AIMS scores were positively correlated with PANSS total scores (r = 0.348, p = 0.032), negative scores (r = 0.458; *P* = 0.004), and SAS scores (r = 0.629; *P*<0.001). The total gray matter volumes of normal control, schizophrenia patients with and without TD were 0.651±0.062, 0.620±0.078, 0.603±0.064 liter, respectively. The schizophrenia patients with TD had significantly smaller gray matter volumes than that of normal control (p = 0.05) (Table 1).

**Table 1:** Demographic/clinical characteristics and grey matter volume among schizophrenia with tardive dyskinesia, schizophrenia without tardive dyskinesia and normal control group.

Demographic variables	Schizophrenia with TD	Schizophrenia without TD	Normal control	p value
	(n = 25)	(n = 25)	(n = 25)	
Age (years)	42.0/11.5	42.0/11.3	41.4/11.8	0.995
Gender (male/female)	8/17	8/17	9/16	0.943
Handedness (left/right)	0/25	0/25	0/25	-
Education (years)	12.1/2.7	12.8/3.2	15.0/2.04	0.001 (schizophrenia with TD<normal control)
GMV (liter)	0.603/0.064^a^	0.620/0.078	0.651/0.062	0.050 (schizophrenia with TD <normal control)
WMV(liter)	0.486/0.045	0.499/0.073	0.508/0.054	0.417
CSFV(liter)	0.245/0.036	0.230/0.050	0.219/0.025	0.059
TIV (liter)	1.336/0.109	1.350/0.172	1.378/0.123	0.531
Age of onset (years)	25.8/6.7	28.6/10.8	-	N.S.
Duration of illness (years)	15.2/7.8	10.7/10.7	-	N.S.
Antipsychotic(CPZ equivalent dose)	428/234.3	450/215.6		N.S.
PANSS				
Total	66.3/12.7	53.9/15.3	-	**0.003**
Positive	15.2/4.9	12.8/4.8	-	N.S.
Negative	17.7/4.6	12.8/4.2	-	**<0.001**
General	33.5/5.3	28.2/8.2	-	**0.012**
AIMS	10.5/4.6	0.1/0.4	-	**<0.001**
SAS	6.8/4.5	0.3/1.0	-	**<0.001**

The variables are demonstrated as means/std. Boldfaced p value indicate significant differences (P<0.05) in appropriate statistical tests. Abbreviation: TD, tardive dyskinesia; N.S., non-significant; GMV, gray matter volume; WMV, white matter volume; CSFV, cerebrospinal fluid volume; TIV, total intracranial volume; PANSS: positive and negative syndrome scale; AIMS, Abnormal Involuntary Movement Scale; SAS, Simpson-Angus Scale.

a ANOVA, P00.05; Post-hoc (Tukey): TD < Normals.

### Whole brain voxel-based morphometry and regional basal ganglia volume results

**Figure 1:**
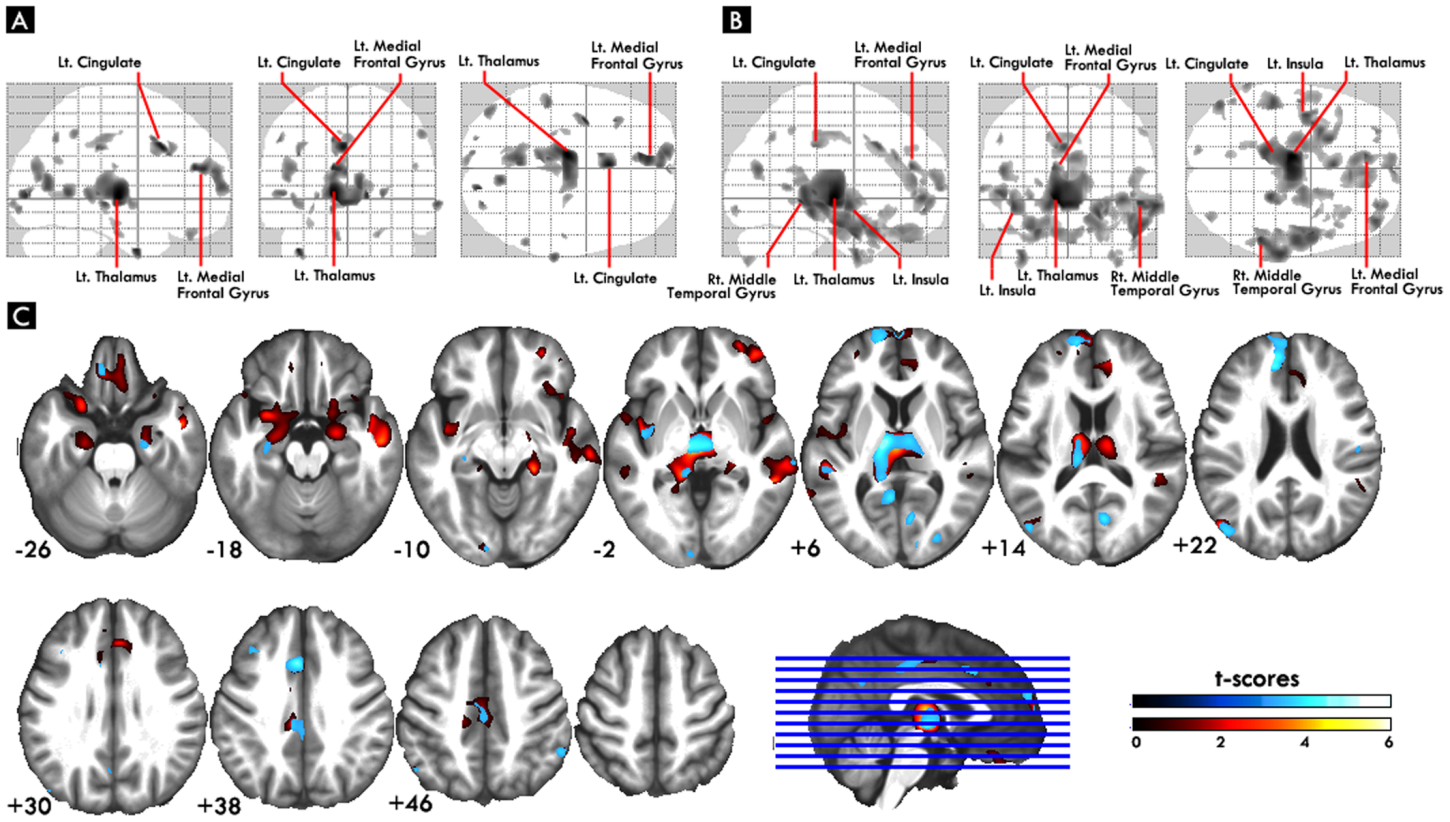
Regional gray matter volume atrophy pattern between disease groups and normal control group.

**Figure 2:**
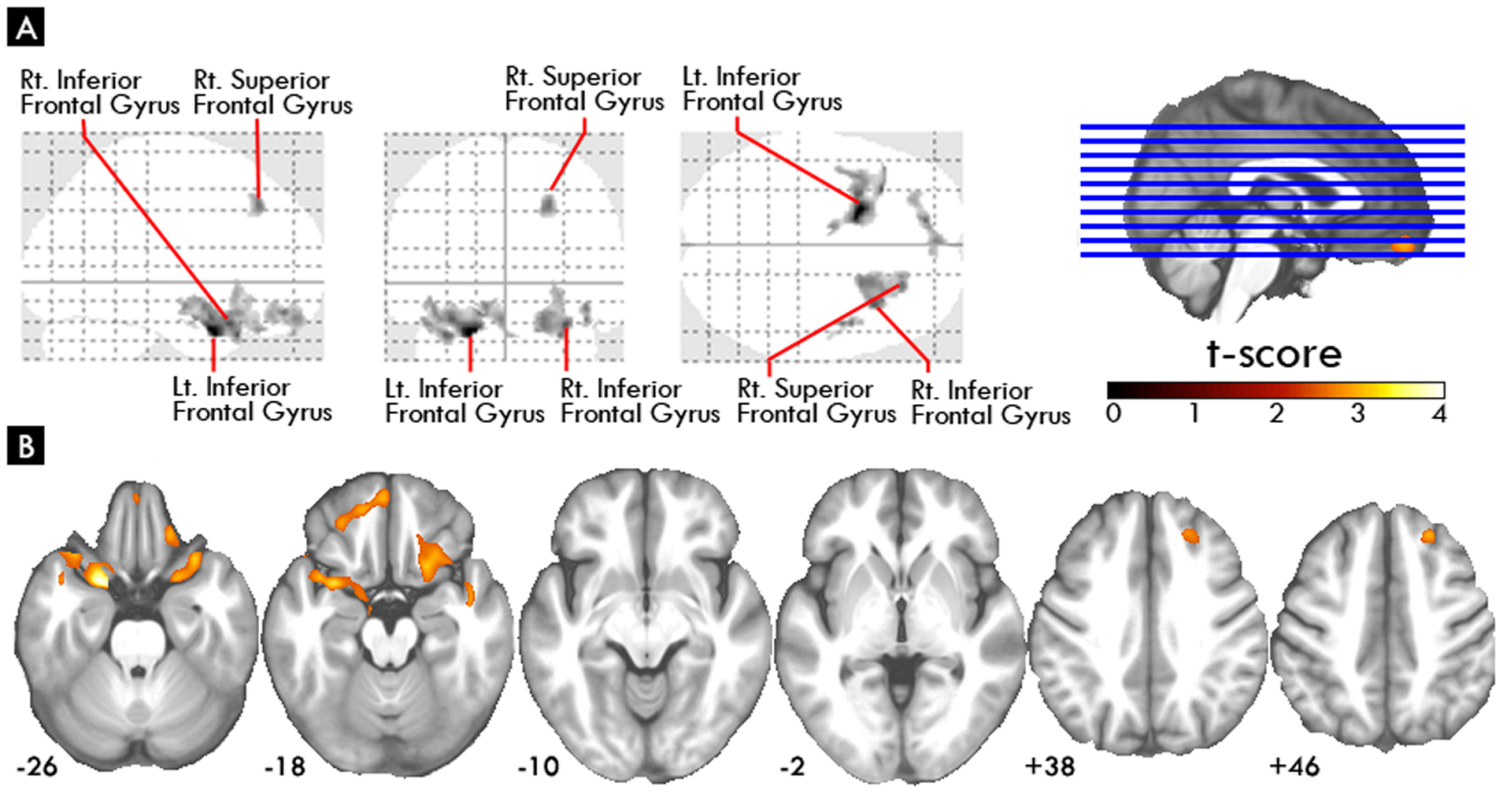
Regions of reduced gray matter volume in schizophrenia with tardive dyskinesia group compared with schizophrenia without tardive dyskinesia group.

As compared to healthy subjects, both groups of schizophrenia patients showed reduced volumes in widespread cortical areas (such as frontal, temporal, parietal and also occipital cortices), insula, thalamus, parahippocampus and cerebellum, but the patients with TD have more gray matter deficits in frontal and medial temporal areas (Figure 1). As for the direct comparisons between schizophrenia with and without TD, the schizophrenia with TD group showed the most significantly reduced volume mostly at the bilateral inferior frontal gyrus, and right superior frontal gyrus (Table 2, and Figure 2). As for the basal ganglia, even after lowering statistical thresholds to an uncorrected p-value of 0.001, there was still no statistical significance in the basal ganglia when directly comparing between schizophrenia patients with and without TD.

**Table 2:** Gray matter anatomical regions with significant volume reduction in Schizophrenia with tardive dyskinesia group compared with Schizophrenia without tardive dyskinesia group.

MNI atlas coordinates			Voxels size	Anatomical Region	Nearest Brodmann Area	Regional GMV Mean (SD)		*Z-Score*
X	Y	Z				SCH w/o TD	SCH w TD	
**Atrophy regional GM volumes in TD vs. SCH**								
−21	10	−27	752	Left Inferior Frontal Gyrus	Brodmann area 47	0.283 (0.046)	0.249 (0.041)	3.98
34	18	−22	159	Right Inferior Frontal Gyrus	Brodmann area 47	0.05 (0.008)	0.044 (0.008)	3.42
24	36	42	64	Right Superior Frontal Gyrus	Brodmann area 8	0.028 (0.005)	0.024 (0.004)	3.26
22	11	−23	167	Right Inferior Frontal Gyrus	Brodmann area 47	0.074 (0.012)	0.065 (0.011)	3.23

The regional gray matter volumes were demonstrated as means (std). The unit of regional gray matter volume is cm^3^. Statistical criteria of the table: uncorrected p-value <0.001 with extended voxel threshold of 50. Abbreviations: MNI, Montreal Neurological Institute; GMV, gray matter volume; SCH w/o TD, schizophrenia without tardive dyskinesia group; SCH w TD, schizophrenia with tardive dyskinesia group.

### Correlations of clinical evaluations and regional gray matters changes in schizophrenia with/without TD patients

**Figure 3:**
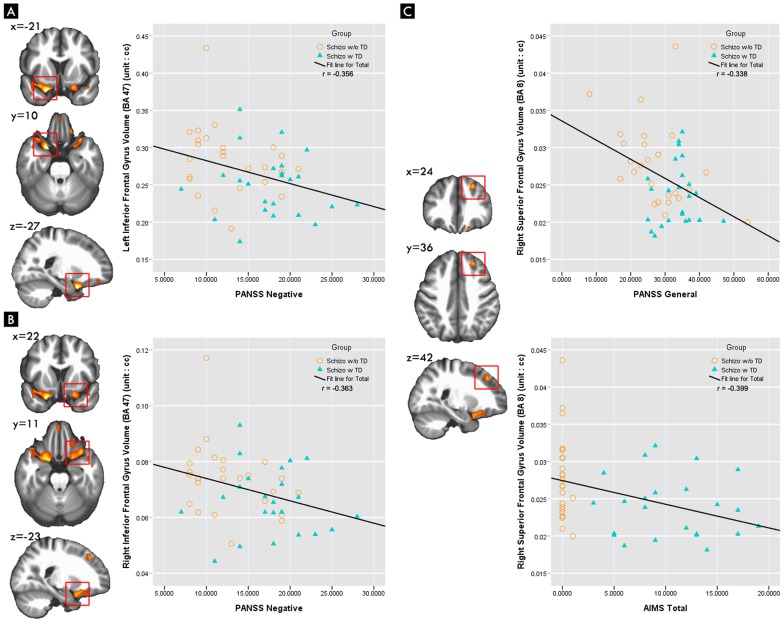
The relationship between clinical evaluations and regional gray natter reduction in disease groups.

The bilateral inferior frontal gyrus were correlated negatively with PANSS negative scale scores (right side r = −0.363, p = 0.014; left side r = −0.356, p = 0.016). The right superior frontal gyrus, which correlated negatively with AIMS scores in a statistical significance after correcting for multiple comparison (r = −0.399, p<0.001); as well as with PANSS general score (r = −0.338, p<0.05), PANSS negative scale scores (r = −0.268, p = 0.075) and SAS scores (r = −0.280 p = 0.063) in a trend significance. (Table 3, Figure 3).

**Table 3:** Partial pearson correlation coefficients between clinical evaluations and reduced gray matter volume derived from the comparison of Schizophrenia with and without tardive dyskinesia groups.

Gray Matter Anatomical Region ( Brodmann Area)	PANSS				AIMS	SAS
	Total	Negative	Positive	General		
Left Inferior Frontal Gyrus (47)	−0.135	−**0.356** a	0.110	−0.087	−0.221	−0.237
Right Inferior Frontal Gyrus_1 (47)	−0.033	−0.252**^c^**	0.172	−0.064	−0.215	−0.160
Right Inferior Frontal Gyrus_2 (47)	−0.125	−**0.363** a	0.078	−0.090	−0.230	−0.140
Right Superior Frontal Gyrus (8)	−0.182	−0.268**^c^**	0.056	−**0.338** a	−**0.399^b,^** *	−0.280**^c^**

Right Inferior Frontal Gyrus_1 indicated the significant cluster which maximum t value located in 34,18, −22 ; Right Inferior Frontal Gyrus_2 indicated the significant cluster which maximum t value located in 22,11, −23. Abbreviation: PANSS: positive and negative syndrome scale; AIMS, Abnormal Involuntary Movement Scale; SAS, Simpson-Angus Scale.

a p-value<0.05; ^b^ p-value<0.01; ^c^ p-value<0.10.

* statistically significant, corrected-p<0.0125.

### Associations between demographic/clinical variables and GM volume changes

The results from stepwise multiple regression analysis showed that the GM volume change of left inferior frontal gyrus (F = 11.79, P<.001) was best predicted by TIV (β = .534), followed by PANSS negative (β = −.490) and PANSS positive scores (β = .362). The GM volume change of the first right inferior frontal cluster (F = 9.28, P<.001; for Right Inferior Frontal Gyrus_1 in Table 3) was best predicted by TIV (β = .433), followed by PANSS negative (β = −.291). That of the second right inferior frontal cluster (F = 8.93, P = .004; for Right Inferior Frontal Gyrus_2 in Table 3) was best predicted by TIV (β = .399). The right superior frontal GM volume change (F = 14.17, P<.001) was best predicted by TIV (β = 0.493), followed by AIMS total scores (β = −.346). Taken together, our results indicated that schizophrenia with TD had reduced GM volume in bilateral inferior frontal gyri and right superior frontal gyrus, with the latter explained the existence and the severity of the dyskinesia.

## Discussion

Several major findings were found in this VBM study. First, the TD versus non-TD patients, not only with dyskinesia, also demonstrated more extrapyramidal side effect (EPS) and severe psychotic symptoms, specifically the negative and general symptoms. Second, the patients with TD had smaller gray matter volumes in the bilateral inferior frontal gyrus and right superior frontal gyrus, but not in the basal ganglion (even under a less strict statistical threshold). Third, the smaller right superior frontal gyrus correlated negatively with AIMS scores, and smaller bilateral inferior frontal gyrus correlated negatively with the PANSS negative scores in schizophrenia patients with and without TD. The reduced GM volume in the right superior frontal gyrus explained the existence and severity of tardive dyskinesia in schizophrenia.

The pathophysiology of neuroleptic-induced TD remains to be fully understood. The leading hypothesis involves dopamine receptor hypersensitivity, and is supported by neuroleptic-induced EPS as the most important risk factor for TD [1], [51], [52]. There are also mounting evidences showed the patients with TD were associated with more severe clinical psychopathology, especially negative symptoms than those without TD [1], [16], [18], [52]–[55]. Taken together, these evidences indicated the neuromotor disturbances of TD may be an important phenotype of schizophrenia, and involve multiple fronto-striatal circuits regulating limbic and neuromotor behavior in schizophrenia [54], [56]. Our results were consistent with previous studies that patients with TD had more severe psychopathology, and could be a phenotype of schizophrenia. But due to the cross-section design, it is not possible to attribute the findings of gray matter change to TD per se, and to separate TD from the context of the illness of schizophrenia. Our VBM analysis showed reduced gray matter volume in the inferior and superior frontal gyrus in the patients with TD versus those without. The volume of the former was correlated negatively with the severity of negative symptoms, and that of the latter was correlated negatively with the severity of TD symptoms. Previous research evidences have shown that negative symptoms of schizophrenia is related to the abnormality of the superior temporal and inferior frontal gyri and reduced frontal-temporo connectivity [57]. The superior frontal gyrus, altogether with other frontal areas such as inferior frontal gyrus and dorsolateral prefrontal cortex, is implicated in the inhibition of unwanted movements [58].

In our study, the long-held believe of the involvement of basal ganglia in TD cannot be found. The results were different from another recent report by Sarro et al [59]. The possible reason for the different results may be related to the different rating scales. Simpson et al rated the severity of tardive dyskinesia by Tardive Dyskinesia Rating Scale (TDRS) [60]. This rating scale is consisted of 43 items, including 8 items which actually evaluate parkinsonism or dystonia, such as item2: tremor of eyelids, item3: tremor of upper lip, item11: tongue tremor, item 18: retrocollis, item19: spasmodic torticollis, item20: torsion movement (trunk), item37: restless legs, and item42: akathisia. The score of each item ranged from 1 to 6. In Sarro et al’s report, the average score of tardive dyskinesia by TDRS was only 10.13± 7.92. Therefore, the patients with parkinsonism or dystonia could be easily identified as TD subjects in their study, and may lead to the positive findings in basal ganglia [59]. Our rating scale for TD is AIMS; which is a widely used rating scale for TD researches [4], [5], [24]–[42], and the instruction of this scale emphasizes to exclude the parkinsonism or dystonia symptoms. Although the long-held believe of the involvement of basal ganglia in TD cannot be found in the our study, the research evidence showed cortical information is processed in the basal ganglia nuclei, which in turn send projections back via the thalamus to the cortex (corticobasal ganglion loop) [61]. Dopamine dysfunction might play a role in the inhibitory function of the prefrontal cortical area, since several lines of evidence have suggested a link between the dopaminergic system and the prefrontal cortex. In fact, basal ganglia output nuclei, the substantia nigra pars reticulata, and the globus pallidus all project to the prefrontal cortex via specific thalamic nuclei [62]. Electrophysiological studies have also shown many functional similarities between the basal ganglia and the cortical areas to which they are connected, suggesting that a similar activation pattern may be expected in cortical and related basal ganglia areas [63]. Besides, several functional neuroimaging studies of the temporal and ordinal control of movement sequences have shown that the inferior prefrontal gyrus, together with other cortical regions, is heavily implicated in sensorimotor coordination, goal-directed preparatory activity, and motor inhibition [64], [65]. Taken together, our findings suggest that the involvement of the superior and inferior prefrontal gyrus, instead of basal ganglia, in TD could expand our understanding about the pathophysiological mechanisms of dyskinesia.

In addition to the hypothesis of dopamine receptor hypersensitivity, another important pathophysiologic theory of TD involves neurotoxicity [66]. Dopamine is metabolized by monoamine oxidase to dihydroxyphenylacetic acid; a byproduct of this reaction is hydrogen peroxide. Free radicals may be involved in the pathogenesis of TD. The potent antioxidants vitamin E, Vitamin B6, and piracetam have been shown to alleviate the severity of TD in randomized double-blind, placebo-controlled studies [67], [68]. These studies supported the neurotoxicity hypothesis. Furthermore, our research team had found many genetic markers for TD, including dopamine D1 [24],D2 [33], [35], D3 and brain-derived neurotrophic factor [38], melatonin receptor [25], beta-arrestin [28], regulators of G-protein signaling [30],Par-4 [31], N-methyl-D-aspartate receptor [34], endothelial nitric oxide synthase [36], hratioquinone oxidoreductase [37], cytochrome P-450 2D6 [40],and neural nitric oxide synthase gene [41]. These genetic vulnerabilities should influence all brain cortex, and supported our finding that the patients with TD had significantly smaller gray matter volumes than the controls.

### Limitations

Several limitations in the study should be addressed. First, consistent with previous studies, the patients with TD had more severe psychopathology, and could be a phenotype of schizophrenia. But due to the cross-section design, it is not possible to attribute the findings of gray matter change to TD per se, and to separate TD from the context of the illness of schizophrenia. Second, the schizophrenia subjects had illness duration of more than 10 years, it was difficult to get all the medical records of previous medications, including type of antipsychotics, duration, and accumulated dosages; and it’s also difficult to analyze the relatedness between the complicated medications and the gray matter changes. Third, the patients with TD had significantly lower education level than the normal controls, which is to be expected. But this factor may partially contribute to the result that patients with TD had significantly smaller gray matter than the normal controls. Forth, the neurocognitive function was not assessed in the present study. Giving the present results showed patients with TD had significantly smaller gray matter, poorer neurocognitive function could be expected and needed to be investigated in the future study. Finally, generalizations from the study are limited by the small number of subjects. More large-scale studies are required to validate the results, to exclude the possibility of type II error for the negative results of basal ganglion area.

### Conclusion

This VBM study compared schizophrenia patients with TD to those without TD. Instead of basal ganglia, the schizophrenia patients with TD had significantly reduced gray matter mostly at the bilateral inferior frontal gyrus and the right superior frontal gyrus, which correlated with severity of clinical symptoms and involuntary movement, respectively. These results may expand the understanding about the pathophysiologic mechanism of TD.
